# Inhibition of autophagy inhibits the conversion of cardiac fibroblasts to cardiac myofibroblasts

**DOI:** 10.18632/oncotarget.12392

**Published:** 2016-10-01

**Authors:** Shivika S. Gupta, Matthew R. Zeglinski, Sunil G. Rattan, Natalie M. Landry, Saeid Ghavami, Jeffrey T. Wigle, Thomas Klonisch, Andrew J. Halayko, Ian M.C. Dixon

**Affiliations:** ^1^ Department of Physiology and Pathophysiology, Institute of Cardiovascular Sciences, Rady Faculty of Health Sciences, Max Rady College of Medicine, University of Manitoba, Winnipeg, Manitoba, Canada; ^2^ Department of Human Anatomy and Cell Science, Basic Medical Sciences Building, Rady Faculty of Health Sciences, Max Rady College of Medicine, University of Manitoba, Winnipeg, Manitoba, Canada; ^3^ Children's Hospital Research Institute of Manitoba, John Buhler Research Centre, Rady Faculty of Health Sciences, Max Rady College of Medicine, University of Manitoba, Winnipeg, Manitoba, Canada; ^4^ Department of Physiology and Pathophysiology, Internal Medicine and Pediatrics and Child Health, Rady Faculty of Health Sciences, Max Rady College of Medicine, University of Manitoba, Winnipeg, Manitoba, Canada; ^5^ Department of Biochemistry and Medical Genetics, Institute of Cardiovascular Sciences, Rady Faculty of Health Sciences, Max Rady College of Medicine, Rady Faculty of Health Sciences, University of Manitoba, Winnipeg, Manitoba, Canada

**Keywords:** cardiac fibroblast, myofibroblast, phenoconversion, autophagy, cardiac fibrosis

## Abstract

The incidence of heart failure with concomitant cardiac fibrosis is very high in developed countries. Fibroblast activation in heart is causal to cardiac fibrosis as they convert to hypersynthetic cardiac myofibroblasts. There is no known treatment for cardiac fibrosis. Myofibroblasts contribute to the inappropriate remodeling of the myocardial interstitium, which leads to reduced cardiac function and ultimately heart failure. Elevated levels of autophagy have been linked to stress-induced ventricular remodeling and other cardiac diseases. Previously, we had shown that TGF-β_1_ treatment of human atrial fibroblasts both induced autophagy and enhanced the fibrogenic response supporting a linkage between the myofibroblast phenotype and autophagy. We now demonstrate that with *in vitro* culture of primary rat cardiac fibroblasts, inhibition of autophagy represses fibroblast to myofibroblast phenoconversion. Culturing unpassaged cardiac fibroblasts for 72 hours on plastic tissue culture plates is associated with elevated α-smooth muscle actin (α-SMA) expression. This activation parallels increased microtubule-associated protein 1A/1B-light chain 3 (LC-3β II) protein expression. Inhibition of autophagy with bafilomycin-A1 (Baf-A1) and chloroquine (CQ) in cardiac fibroblasts significantly reduces α-SMA and extracellular domain A fibronectin (ED-A FN) protein *vs* untreated controls. Myofibroblast cell migration and contractility were significantly reduced following inhibition of autophagy. These data support the possibility of a causal link between cardiac fibroblast-to-myofibroblast phenoconversion and autophagy.

## INTRODUCTION

In response to the loss of heart muscle following myocardial infarction (MI), the cardiac interstitium is significantly remodeled and contributes to the pathogenesis of heart failure [1–3]. Following MI, the infarct zone heals, but continued, excessive activation of resident cardiac fibroblasts eventually culminates in global cardiac fibrosis with both impaired lusitropic and inotropic function [4]. Normal healthy myocardium is not populated by myofibroblasts [5] and the hallmark of cardiac fibrosis is fibroblast activation to myofibroblasts [5, 6]. While cardiac fibroblasts are relatively quiescent cells that contribute little to matrix remodeling or wound healing, phenoconverted myofibroblasts persist within the infarcted myocardium and contribute to excessive ECM deposition [7–9]. Myofibroblasts are contractile cells that express α-smooth muscle actin (α-SMA), which, in combination with the appearance of stress fibres, is a reliable marker for the myofibroblast phenotype [5, 10]. Extracellular domain A fibronectin (ED-A FN) is also expressed in the myofibroblast and has been noted as an important biomarker for the activated phenotype. [5]. Understanding the mechanisms that activate the conversion of cardiac fibroblasts into hypersecretory, contractile cardiac myofibroblasts is an important topic for investigation [11, 12].

Autophagy is a highly-conserved catabolic process that appears to govern several cardiac pathologies [2, 13]. In our previous study we have used human atrial fibroblasts, and found that the onset of autophagy and cardiac fibrosis are sequentially linked [14]. As we found that TGF-β_1_ treatment of human atrial fibroblasts caused a parallel induction of fibrogenesis and autophagy [14], we have now investigated the effect of autophagy inhibition on rat cardiac fibroblasts and its conversion to myofibroblasts. The implication of autophagy in the induction of the fibrotic response opens a novel area for investigation of therapeutic targets for amelioration of cardiac fibrosis to reduce the risk of heart failure.

The degree of autophagic induction influences the adaptive or maladaptive changes in cardiac tissue [15]. Previous work indicates a putative balance between adaptive and maladaptive autophagic induction and using autophagy inhibitors may shed light on the role of autophagy in the associated pathological signaling processes [13, 16]. Studies regarding heart failure and autophagy have revealed autophagosomes within the myocardium in ischemia/reperfusion models [17, 18]. Several studies have exploited autophagy inhibition in the diseased heart using lysosomotropic agents such as bafilomycin-A1 (Baf-A1) and chloroquine (CQ) [19–21]. Autophagy has been involved in epithelial to mesenchymal transition (EMT) and mesenchymal to epithelial transition (MET) [22, 23] and these events contribute to cell differentiation, wound healing, stem cell proliferation and cancer progression [23]. Li *et al*. have shown that starvation-induced autophagy promotes EMT through TGF-β/Smad signaling in hepatocarcinoma cells and that inhibition of autophagy in these cells with CQ or Autophagy-related gene-3/7 (ATG3-ATG7) siRNA treatments suppresses EMT and decreases cancer cell invasiveness [22]. Thus, the link between autophagy and cell differentiation exists.

Despite these findings, the possibility that autophagy promotes fibroblast activation and phenoconversion in unpassaged cardiac fibroblasts has not been explored. Here we test the hypothesis that autophagy activates phenoconversion of cardiac fibroblasts to myofibroblasts, and that inhibition of the autophagy abrogates this event.

## RESULTS

### Detection of the myofibroblast phenotype and accumulation of lipidated LC-3β II

**Figure 1:**
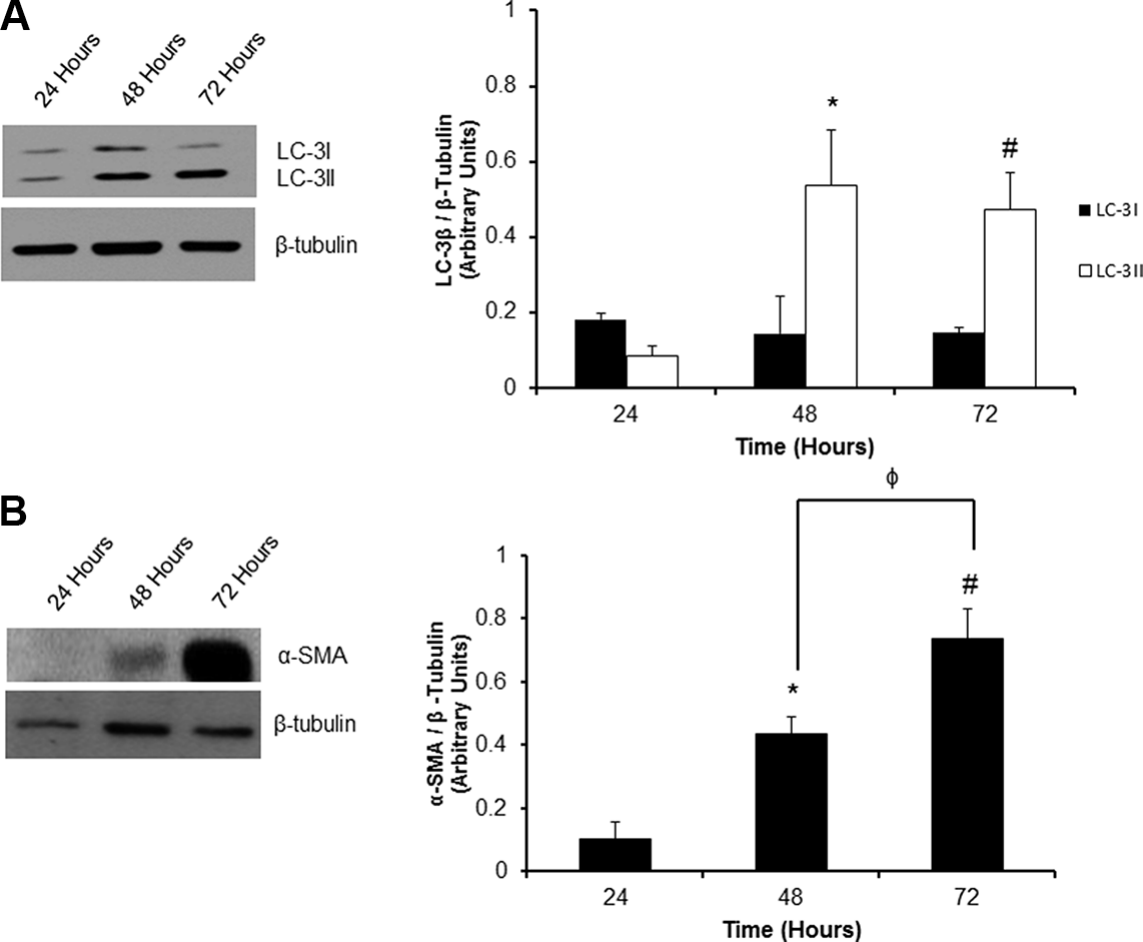
Temporal activation of autophagy and phenoconversion in P0 adult rat cardiac fibroblasts (Panel **A**) There was a significant increase in the level of the autophagosome marker, LC-3β II at 48 and 72 hours post-plating on a non-compressible plastic substrate vs. 24 hours. (Panel **B**). Western blot analysis for the myofibroblast marker α-SMA showed a significant increase 48 and 72 hours after plating when compared to 24 hour controls. Data are mean ± SEM (*n* = 3) (*P < 0.05 24 hours *vs*. 48 hours; ^#^*P* < 0.05 24 hours *vs*. 72 hours; ^φ^*P* < 0.05 48 *vs*. 72 hours).

The time-dependency of autophagy induction by cultured P0 cardiac fibroblasts was determined by measuring LC-3β I and lipidated LC-3β II levels at 48 and 72 hours post-plating (Figure 1A). We found a 6-fold induction of lipidated LC-3β II protein levels as early as 48 hours after plating as compared to the 24 hour control. This trend continued out to 72 hours post-plating where there was still a significant (^#^*P* < 0.05) 5.5-fold increase of LC-3β II levels compared to the 24 hour control.

Concomitantly, to estimate the extent of fibroblast to myofibroblast phenocoversion of unpassaged cardiac fibroblasts over time, we tracked the expression of α-SMA over 72 hours post-plating. Western blot analysis revealed a significant, 4.3- and 7.4-fold increase in α-SMA protein expression at 48 and 72 hours post-plating as compared to the 24 hour control group, respectively (Figure 1B).

### Inhibition of autophagy in P0 primary cardiac fibroblasts

**Figure 2:**
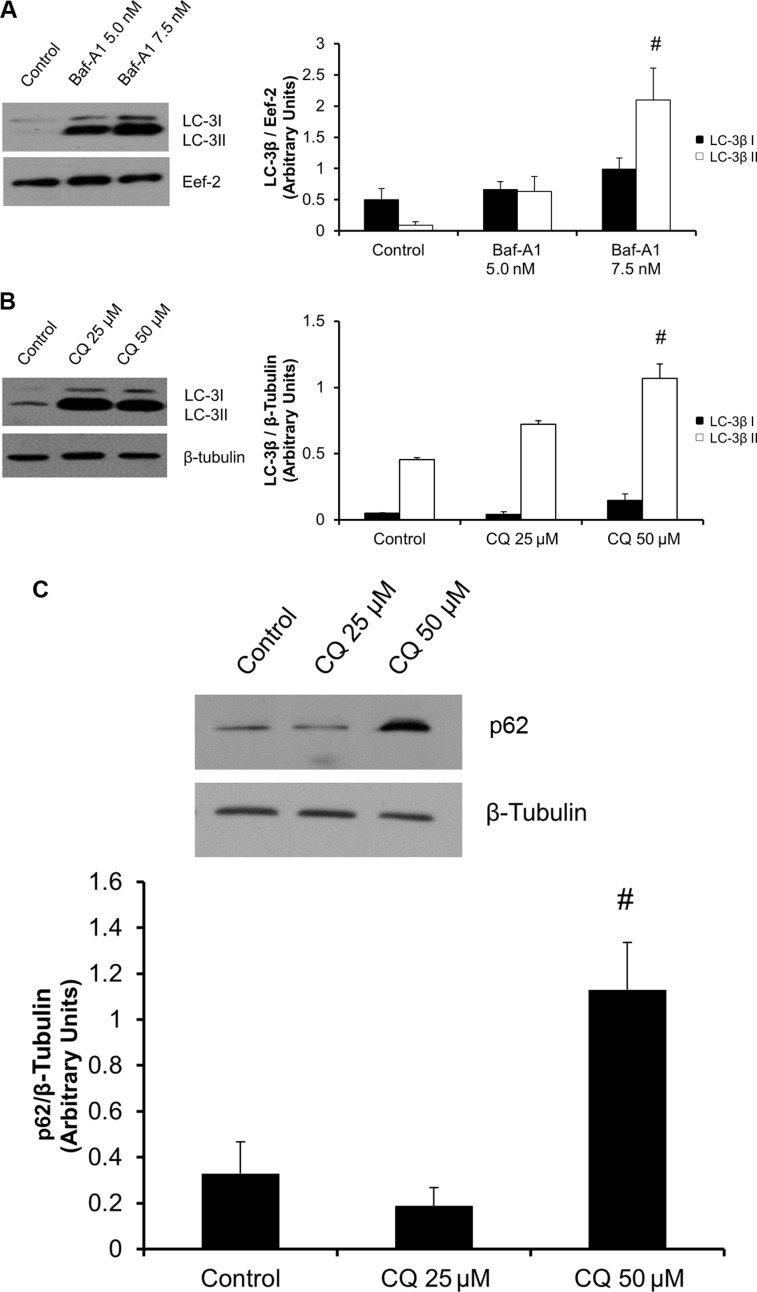

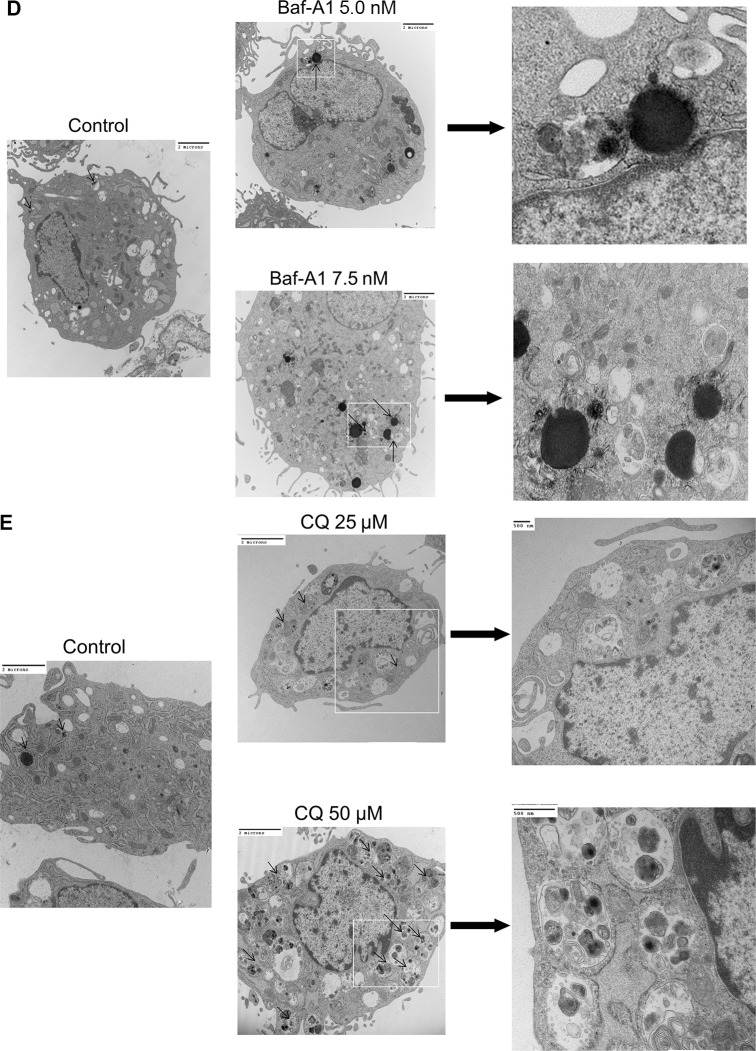
Baf-A1 and CQ treatment inhibits autophagy in P0 cardiac fibroblasts (Panel **A** and **B**) 48 hours of Baf-A1 treatment (5.0 nM and 7.5 nM) showed no significant change in LC-3β I protein expression. However there was a significant increase in the expression levels of LC-3β II with 7.5 nM Baf-A1 *vs*. untreated controls. Cells treated with CQ (25 μM and 50 μM) for 48 hours demonstrated increased accumulation of LC-3β II *vs*. untreated controls. The 50 μM CQ treatment group was significantly increased *vs*. control while the 25 μM group was trending upward but did not reach significance. (Panel **C**) p62 protein levels were significantly increased following CQ (50 μM) treatment *vs*. untreated control. (Panels **D** and **E**). Ultrastructure of rat ventricular cardiac fibroblast. TEM confirms the presence of accumulated autophagosomes as compared to time matched controls in those cells treated with Baf-A1 (5.0 nM and 7.5 nM; D) and CQ (25 μM and 50 μM; E). The magnification for all images are 5000 and 10,500, 19,000 or 30,000. The scale is shown on each image. Data are mean ± SEM (*n* = 3–4) (^#^*P* < 0.05 control *vs*. 7.5 nM Baf-A1 and 50 μM CQ).

To investigate the relationship between autophagy and phenoconversion of primary cardiac fibroblasts, we inhibited the autophagic flux with either Baf-A1 or CQ. We then assayed for the levels of LC-3β I and lipidated LC-3β II. Both Baf-A1 and CQ treatment groups showed a significant (^#^*P* < 0.05) increase in LC-3β II lipidation *vs*. controls (Figure 2A and 2B). Furthermore, p62 has been identified as a cargo protein which tags and brings protein aggregates to the autophagosomes [24]. Lysosomal degradation of autophagosomes leads to a decrease in p62 levels during autophagy. Conversely, autophagy inhibitors can lead to the accumulation of p62 protein levels [24]. Thus, we investigated the expression of p62 in cardiac fibroblast phenoconversion. Regarding CQ treatment, we saw a significant, 3.4-fold (^#^*P* < 0.05) increase in p62 levels in cells treated with 50 μM CQ, as compared to untreated controls (Figure 2C).

Transmission electron microscopy (TEM) studies were performed to assess for autophagosomes in both Baf-A1 and CQ treated cells. TEM confirmed the presence and accumulation of autophagosomes in cells treated with both autophagy-inhibiting drugs (Figure 2D and 2E, black arrows indicate accumulated autophagosomes).

### Inhibition of autophagy in unpassaged P0 cardiac fibroblasts inhibits the myofibroblast phenotype

**Figure 3:**
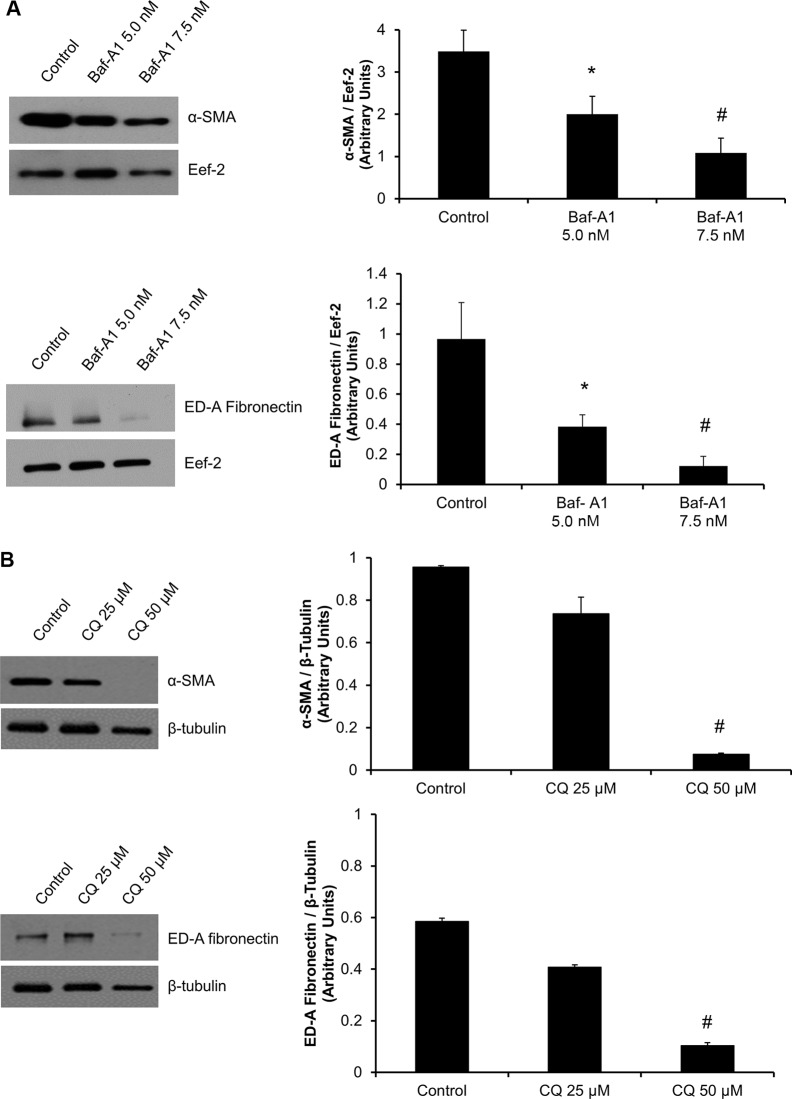

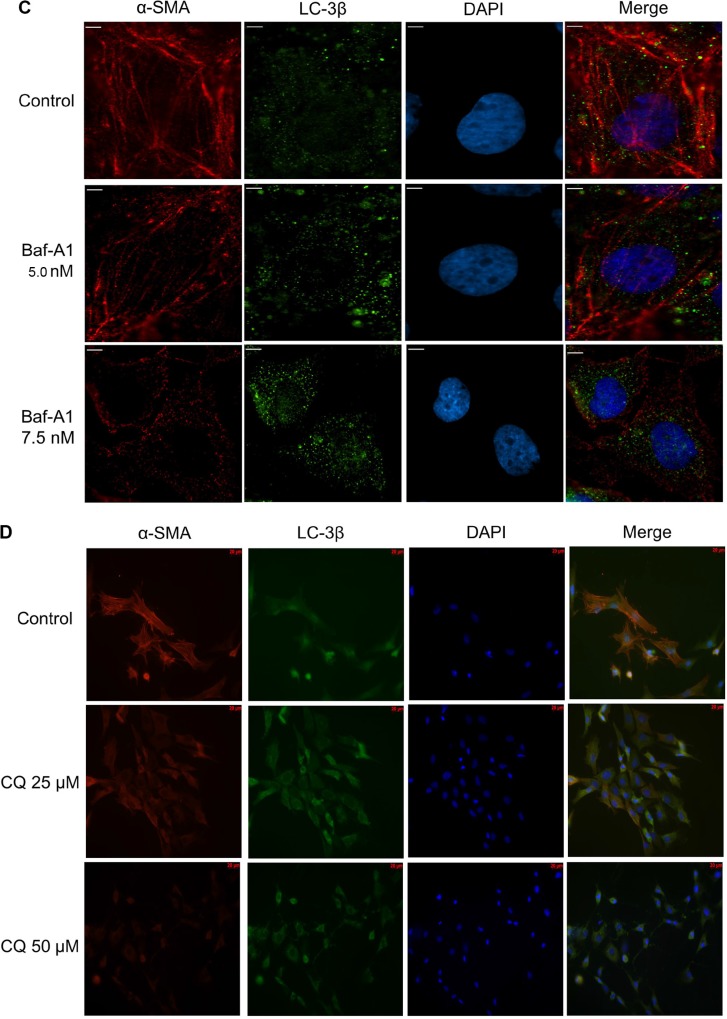
Inhibition of autophagy reduces expression of key cardiac myofibroblast markers Western blot analysis of P0 cardiac fibroblasts treated with autophagy inhibitors show decreased expression levels for ED-A FN and α-SMA. (Panel **A**) 48 hours of Baf-A1 (5.0 nM and 7.5 nM) treatment resulted in a significant reduction in α-SMA (40% and 70%) and ED-A FN (35% and 50%) levels respectively. (Panel **B**) 50 μM CQ treatment for 48 hours shows a significant decrease in α-SMA and in ED-A FN levels to near undetectable levels. (Panels **C** and **D**). Immunofluorescence staining of P0 cardiac fibroblast shows decreased α-SMA protein expression increased LC-3β punctate using 5 nM and 7.5 nM Baf-A1 as well as 25 μM and 50 μM CQ *vs.* untreated control cells. Data are expressed as mean ± SEM (*n* = 3–4) (*P < 0.05 control *vs.* 5.0 nM Baf-A1; ^#^*P* < 0.05 control *vs.* 7.5 nM Baf-A1, 50 μM CQ).

We have noticed a time-dependent induction of the myofibroblast phenotype in P0 cells plated on hard plastic, which correlates with increased levels of lipidated LC-3β II. This prompted us to assess the effects of autophagy inhibition on the expression of myofibroblast markers. Western blot analysis revealed a significant (*^#^*P* < 0.05) reduction in the key myofibroblast markers, α-SMA and ED-A FN, with both Baf-A1 and CQ treatment (Figure 3A and 3B). Both autophagy inhibitors abrogated the myofibroblast phenotype.

Further evidence linking autophagy to the myofibroblast phenotype was observed by immunofluorescence staining for α-SMA protein expression. We detected a reduction in α-SMA stress fibre formation and an increase in LC-3β II punctate staining for both Baf-A1 and CQ treatment groups *vs.* controls, supporting our Western blot data (Figure 3C and 3D).

### Inhibition of autophagy in unpassaged P0 cardiac fibroblasts inhibits myofibroblast function

**Figure 4:**
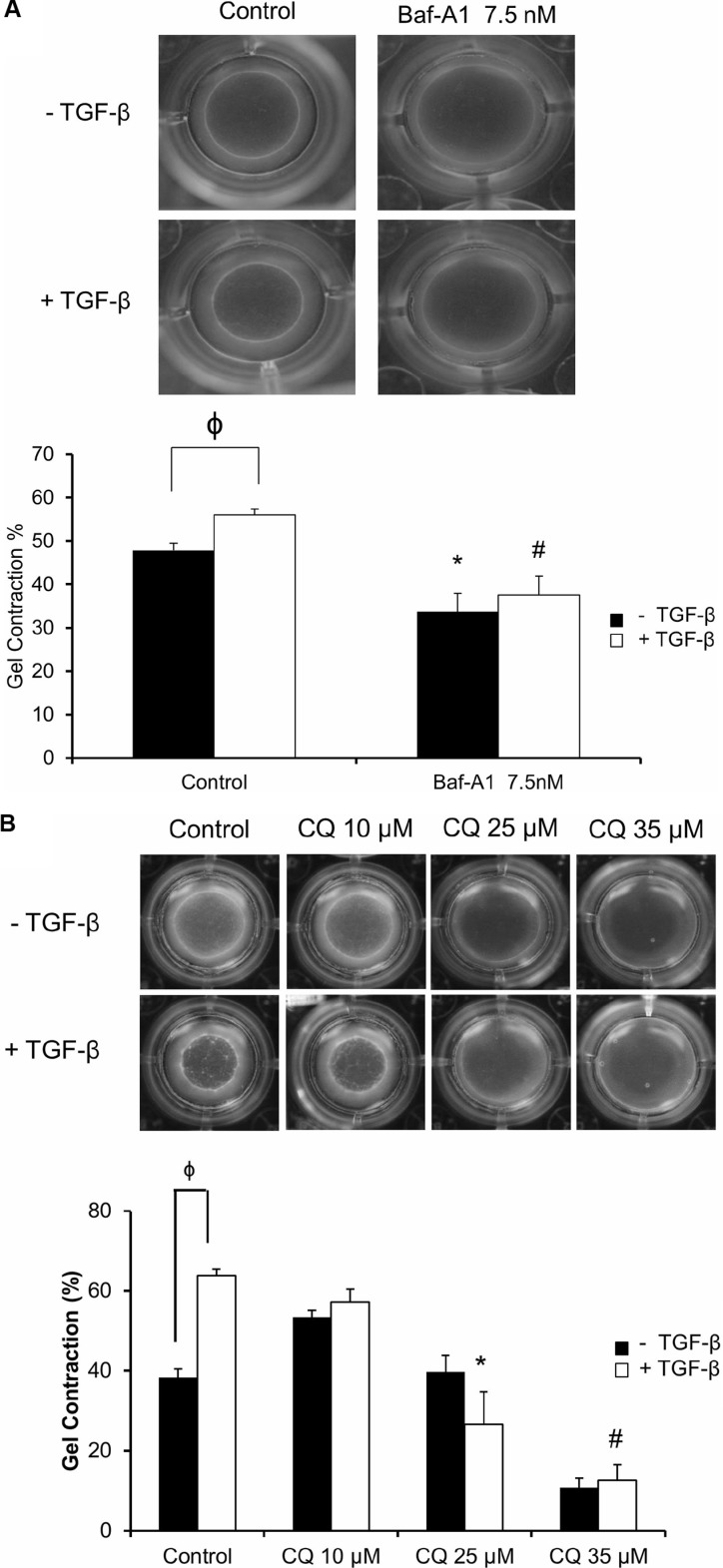

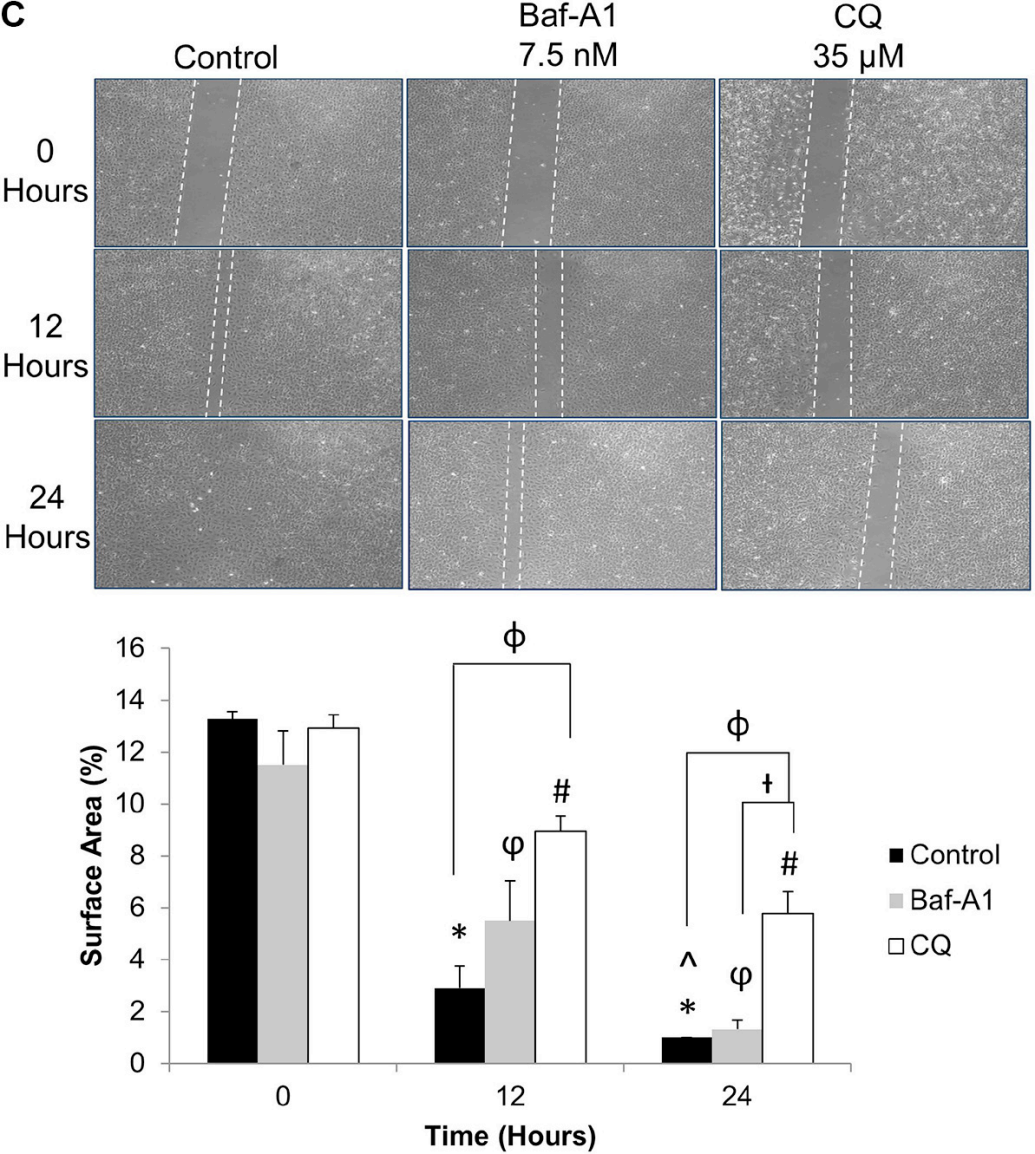
Effect of autophagy inhibition on cellular contractility and migration of P0 cardiac fibroblasts (Panels **A** and **B**) Gel contraction assay of P0 cardiac fibroblasts treated with 7.5 nM Baf-A1 (4A) and 10–35 μM CQ (4B) with and without 10 ng/ml TGF-β_1_ for 48 hours. Solid black bars represent untreated and white bars represent TGF-β_1_ treated cells. Both gel images and histographic representation of the data demonstrate that 7.5 nM Baf-A1 and 35 μM CQ significantly inhibited fibroblast mediated gel contraction *vs.* untreated controls Baf-A1 + TGF-β_1_ and CQ + TGF-β_1_ treatment also shows a significant decrease in contraction as compared with TGF-β_1_ treated positive control. Data are mean ± SEM (*n* = 3–4) (^ϕ^ *P* < 0.05 comparison of TGF-β_1_ (-) *vs.* TGF-β_1_ (+) groups; *P < 0.05 for control *vs.* 7.5 nM Baf-A1 treatment, and control + TGF-β_1_ *vs.* 25 μM CQ + TGF-β_1_; ^#^*P* < 0.05 for control + TGF-β_1_ *vs.* 7.5 nM Baf-A1 + TGF-β_1_ and control + TGF-β_1_ *vs.* 35 μM CQ + TGF-β_1_). (Panel **C**) Scratch assay to assess cellular migration of cardiac fibroblasts in the presence and absence of 7.5 nM Baf-A1 or 35 μM CQ. The solid black bars represent untreated controls, grey bars Baf-A1 treated and white bars represent CQ treated. Migration was gauged by the gradual population with cells migrating into the cell free zone on the glass slide over time. Migration of fibroblasts in control (untreated cells) and in cells treated with 7.5 nM Baf-A1 or 35 μM CQ are depicted in the images. 12 hours after the initiation of the assay we found a significant decrease in migration in CQ treated cells when compared to untreated controls. Similarly, after 24 hours, CQ treated cells were found to migrate more slowly than untreated controls. Control groups were ~ 100% confluent at 24 hours. (*P < 0.05 for 0 hours *vs.* 12 hours and 24 hours in untreated controls; ^φ^*P* < 0.05 for 0 hours *vs.* 12 hours and 24 hours in 7.5 nM Baf-A1 treated groups; ^#^*P* < 0.05 for 0 hours *vs.* 12 and 24 hours in 35 μM CQ treatment groups; ^†^*P* < 0.05 for Baf-A1 *vs.* CQ 35 μM at 24 hours; ^ϕ^*P* < 0.05 for untreated control *vs.* CQ 35 μM, treated groups within the same time point). Data are mean ± SEM (*n* = 3–4).

After observing an effect of autophagy on the myofibroblast phenotype, we then sought to investigate the effects of autophagy inhibition on myofibroblast function by measuring cell contractility. Using a collagen gel-based contraction assay, we found that both Baf-A1 (7.5 nM; Figure 4A) and CQ (25 and 35 μM; Figure 4B) significantly (^#^*P < 0.05) inhibited collagen gel contraction as compared to untreated and TGF-β_1_ treated controls after 72 hours. TGF-β_1_ stimulated cells which were not treated with autophagy inhibitors served as a positive control for gel contraction, as previously described [25].

To assess fibroblast migration and its relationship to autophagy, we performed scratch assays in the presence of autophagy inhibitors and measured the time required for fibroblasts to fill the empty space (*i.e.* migrate). There was a noticeable reduction in fibroblast migration as early as 12 hours *vs.* 0 hour control in both Baf-A1 and CQ treated cells. This effect continued up to the 24 hour in CQ treated cells, when compared with the untreated controls that had filled the denuded area. In the CQ treated (35 μM) group, we observed an approximately 3- and 6-fold reduction in cell migration after 12 and 24 hours, respectively. However the Baf-A1 *vs.* untreated control did not reach significance. (Figure 4C; *P < 0.05 0 hour *vs.* 12 hour and 24 hour; ^ϕ^*P* < 0.05 0 hour *vs.* 12 and 24 hour Baf-A1 7.5 nM; ^#^*P* < 0.05 0 hour *vs.* 12 and 24 hour 35 μM CQ; ^†^*P* < 0.05 Baf-A1 7.5nM *vs*. CQ 35 μM; ^φ^*P* < 0.05 control *vs.* CQ 35 μM). We therefore conclude that autophagy may contribute to fibroblast migration.

### Effect of autophagy on mitogen-activated protein kinases (MAPK) signaling

**Figure 5:**
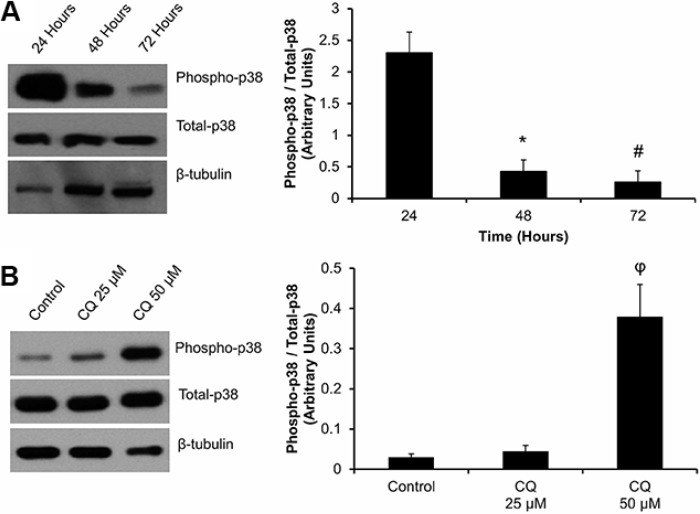
Effect of cell plating and CQ treatment on p38-MAPK phosphorylation in P0 primary cardiac fibroblasts: (Panel A) Phosphorylation of p38-MAPK was detected at 24, 48 and 72 hours after plating of P0 cardiac fibroblasts onto a non-compressible plastic substrate. Total p38-MAPK was also analysed. Phospho-p38 (P-p38) expression was significantly decreased after 48 and 72 hours of culture when compared to 24 hour controls. (Panel **B**) Western blot images indicate an increase in phosphorylation of p38-MAPK in the presence of 50 μM CQ treated for 48 hours, as compared to untreated controls. Lane protein loading was normalized using β-tubulin. Data are mean ± SEM (*n* = 3) (*P < 0.05 applies to the comparisons of 24 hours plated cells *vs.* 48 hours plating; ^#^*P* < 0.05 applies to the comparison of 24 hours plating *vs.* the 72 hours plated group; ^φ^*P* < 0.05 for control *vs.* 50 μM CQ).

As MAPK signaling has been shown to play a role in the myofibroblast phenotype, we assayed for the activation (phosphorylation) of p38 in cardiac fibroblasts plated over 72 hours (Figure 5A). We found that 48 and 72 hours post-plating, there was a significant, (^#^*P < 0.05 *vs.* 24 hour) 80 to 90% reduction in the ratio of phosphorylated-p38 (phospho-p38):total p38 levels. We inhibited autophagy in these cells with CQ treatment and reassessed these cells for p38 activation (Figure 5B). In cells treated with 50 μM CQ we found a 12-fold increase (*P < 0.05) in phospho-p38:total p38 levels after 48 hours of treatment, which were comparable to that of unpassaged P0 fibroblasts 24 hours after plating (Figure 5A).

## DISCUSSION

In the current study, we demonstrate a link between autophagy and the activation of unstimulated P0 cardiac fibroblasts. This activation, and subsequent phenoconversion, of quiescent fibroblasts to the hypersynthetic myofibroblast phenotype is a hallmark event of myocardial would healing, a process which underpins the fibrotic remodeling response of cardiac tissue following injury [26]. During the normal wound healing in the post-MI heart, hypersynthetic myofibroblasts acutely produce more ECM proteins to repair the damaged myocardium [5, 26]. Of the numerous mechanisms described to regulate this process, TGF-β_1_ is arguably the most potent pro-fibrotic stimulus [25]. TGF-β_1_ functions *via* the R-Smad/Co-Smad-dependent pathway, and through the Smad-independent p38-MAPK signaling pathway [27]. We have recently shown that autophagy may regulate TGF-β_1_ induction of fibrogenesis in primary human myofibroblasts [14].

In eukaryotic cells, activation of autophagy is linked to enhanced longevity [28], and may also have important cytoprotective effects in fibroblasts. It was recently demonstrated that embryonic fibroblasts isolated from mice overexpressing pro-autophagic ATG5 exhibited higher resistance to cellular stress [29]. Although the specific relationship between autophagy and cardiac fibrosis is under-represented in the literature, autophagy has been extensively studied in cardiomyocyte function and death in the face of pathologic stimuli, with emphasis on remodeling of the myocardium. Apoptotic and non-apoptotic myocyte dropout are important contributors to cardiac remodeling and the pathogenesis of heart failure [30]. Hariharan *et al.* have shown that cardiomyocyte death and pathological remodeling in an I/R injury and pressure overload model of the heart is associated with oxidative stress and increased autophagy [31]. Inhibition of pro-autophagic Beclin-1 was shown to alleviate the effects of chronic remodeling in these two experimental models [31]. Additionally, in a study of mouse and human stellate cells autophagy has been linked to the promotion of liver fibrosis [32]. Conversely, the inhibition of autophagy has been implicated in reduced fibroproliferative events in many tissues [33]. Our present dataset indicates a putative role for autophagy in myofibroblast phenoconversion which may mediate fibroproliferative events in the post-MI heart.

Fibrosis of the myocardium is attended by elevated muscle stiffness and decreased cardiac output. Olsen *et al.* showed that culturing the culture of primary rat hepatic stellate cells on relatively stiff substrate will potentiate their activation to the myofibroblast phenotype, even in cells isolated from TGF-β null mice [34]. Studies on skin and heart tissues indicate that long-term mechanical strain is augmented by scarring and fibrosis [35, 36]. These findings support the premise that alteration in tissue stiffness is associated with progression of tissue fibrosis. Thus, we have expanded our exploration of autophagy as a contributor to the phenoconversion of cardiac fibroblasts by plating them on plastic tissue culture plates.

Plating primary fibroblasts on a non-compressible substrate induces the myofibroblast phenotype within hours of culture [5, 25, 37, 38]. We found that within 48 hours of culture on a stiff (non-compressible) plastic substrate, unpassaged P0 fibroblasts exhibited a significant up-regulation of the key myofibroblast markers α-SMA and ED-A FN that continued out to 72 hours. We found that induction of the myofibroblast phenotype occurred concomitantly with significant upregulation of autophagy, as indicated by increased expression of lipidated LC-3β II 48 and 72 hours post-plating as compared to 24 hour controls. The present work provides information linking autophagy to phenotype conversion to myofibroblast when plated on plain tissue culture plates. This finding agrees with our previously published report which demonstrated that TGF-β_1_ stimulation is causal to the induction of autophagy and enhanced cell activation. Cell activation led to ECM production in both human atrial and rat ventricular cardiac tissues following coronary artery bypass graft (CABG) and post-MI models respectively [12]. As we have discussed above autophagy plays and important role in regulation of cellular phenotype in fibroblasts. Studies by Li *et al.* and others have indicated that autophagy is necessary for phenotype plasticity of hepatocytes and is responsible for hepatic carcinoma cell metastasis through activation of EMT, which includes the activation of TGF-β_1_ signaling [22, 39] This discussion highlights the importance of autophagy in regulation of cellular phenotypes and its function in diseases development.

Autophagy has been described by us [14] and others [40] as a critical cellular event in fibrosing tissues, we set out to describe the mechanisms associated with autophagy and the phenoconversion process. Western blotting for p62 and LC-3β indicated successful inhibition of autophagy, as indicated by increased levels of p62 and LC-3β II levels when using the inhibitory drugs Baf-A1 and CQ [41]. Protein p62 is a cargo protein that binds autophagosomal membrane protein LC-3β and brings p62-containing protein aggregates to the autophagosome [24]. Inhibition of autophagy leads to accumulation of LC-3β II [21] and p62. Using TEM imaging, we found increased accumulation of autophagosomes, which we interpret as inhibition of the completion of autophagy in these cells. We also noted that inhibition of autophagy using Baf-A1 and CQ was correlated with the diminution of the myofibroblast phenotype, as indicated by a significant reduction in α-SMA and ED-A FN protein levels as compared to untreated controls. In addition, we noted that CQ was a more effective inhibitor of myofibroblast phenoconversion than Baf-A1. The current data agree on those of He *et al*. [42] who demonstrated that CQ treatment of hepatic stellate cells in an *in vivo* liver fibrosis model diminished the expression and organization of α-SMA.

The role of autophagy in repressing cardiac myofibroblast function, specifically cell contraction and migration, is not well described. Previous reports, however, have provided insight into the role of autophagy in other cells types [43–45]. Knockout of ATG5 in cardiomyocytes leads to decreased contractile force, whereas loss of ATG7 in skeletal muscle is associated with muscle loss and significantly reduced force production [43]. The current results agree on these previous reports in that inhibition of autophagy reduces myofibroblast contractility. The effects of autophagy on cellular migration has been assessed in HeLa and other transformed cells [40, 46]. Tuloup-Minguez *et al*. [40] showed that knockdown of either ATG3, −5, or −7 in HeLa cells promoted increased cell migration as compared to ATG competent cells. Indelicato *et al*. found that using trifluoperazine, a potent activator of autophagy, could inhibit migration of MDA-MB-231 breast cancer cells [46]. These reports differ significantly from the present study as we demonstrate that inhibition of the autophagy with CQ significantly inhibited myofibroblast migration over 24 hours. The difference between our current data and others may be due to the use of primary, unpassaged cardiac fibroblasts versus immortalized/cancer cell lines. CQ has non-specific inhibitory effects on Toll-like receptor 9 (TLR9), a factor known to be important for cell migration [47]. To support the current data set, CQ treatment of MGC803 gastric cancer cells inhibited cell migration in a dose-dependent fashion [47]. While the reduction of cell motility in our current study was unexpected, this effect may be attributed to CQ-mediated inhibition of TLR-9 function.

We noted a significant decrease in phospho-p38 MAPK expression which occurred in association with the induction of autophagy and the activation of the myofibroblast phenotype 48 hours after plating. Inhibition of autophagy with 50 μM CQ was attended by relatively high expression of phospho-p38 which was comparable to untreated P0 cardiac fibroblasts, 24 hours after plating. We stress that while phosphorylation of p38-MAPK is linked to the inhibition of autophagy, the function and significance of this event remains unknown. A previous study by Fang *et al.* examined the effects of CQ on p38-MAPK signalling pathways in a rat model of hepatic ischemia/reperfusion injury [48]. They noted a significant increase in the phosphorylation of p38 with CQ treatment as early as 6 hours, which continued out to 48 hours post injury; this result is supportive to our current findings.

**Figure 6:**
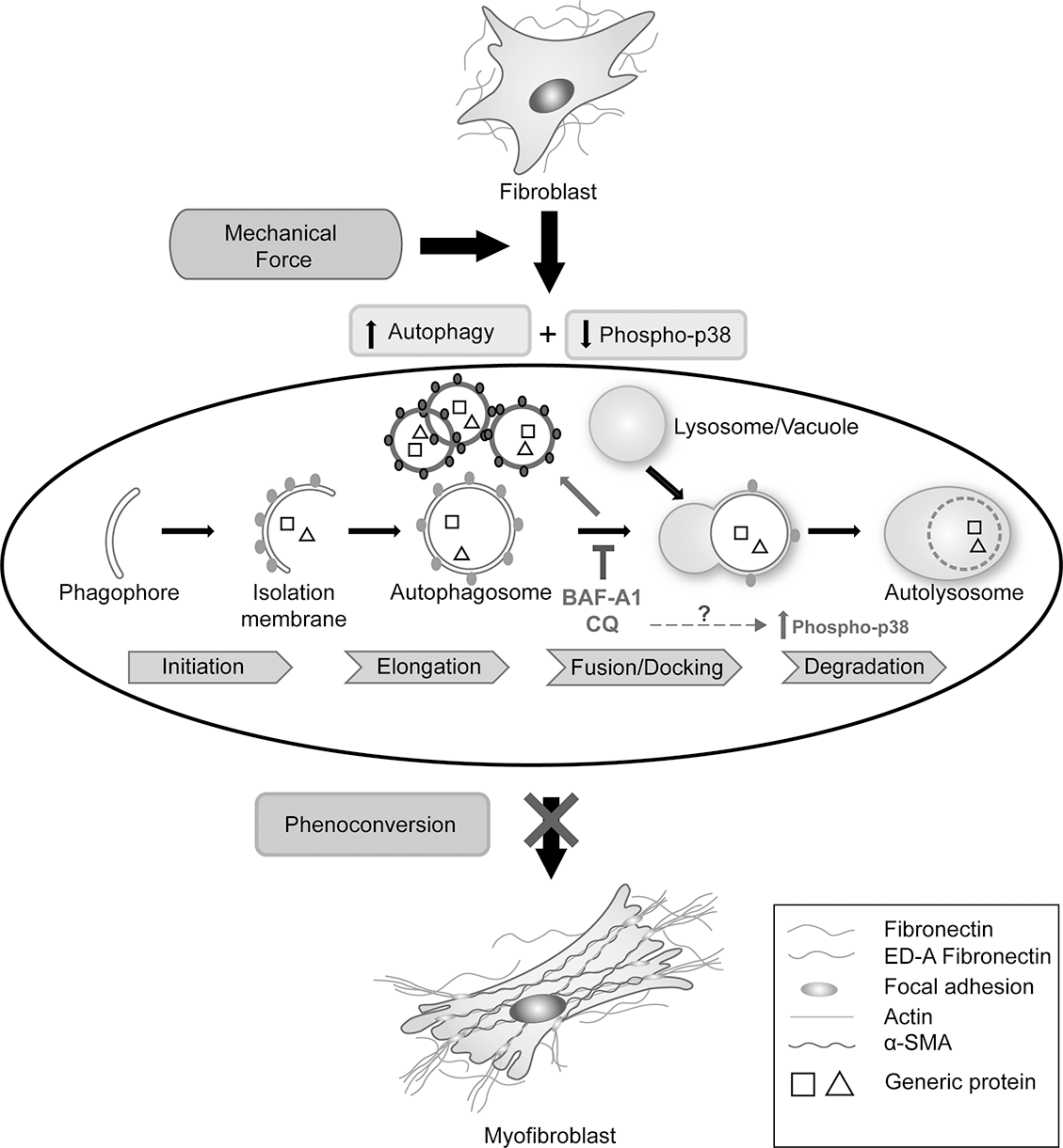
Schematic of the relationship between autophagy and cardiac fibroblast-to-myofibroblast phenoconversion The plating of primary cardiac fibroblasts on a non-compressible plastic substrate is associated with the mechanical induction of autophagy and these events are associated with the activation and phenoconversion to myofibroblasts. The addition of autophagy inhibitory drugs Baf-A1 and CQ blocks the fusion of autophagosomes to lysosomes. Concomitantly, we observed a significant increase in p38-MAPK phosphorylation. Inhibition of autophagy correlates with significant suppression of the myofibroblast phenotype.

A schematic to summarize the effects of plating of primary cardiac fibroblasts, induction of autophagy and the effect of Baf-A1 and CQ on autophagy and fibroblast phenoconversion is shown in Figure 6. We propose that plating of primary cardiac fibroblasts on a non-compressible plastic substratum is associated with the mechanical induction of autophagy and these events correlate with the activation and phenoconversion to cardiac myofibroblasts. Adding autophagy inhibitors blocks the fusion of autophagosomes with lysosomes and correlates with significant suppression of the myofibroblast phenotype. These findings support those of our previously published work [14].

The current results support the suggestion that CQ may attenuate activation of cardiac fibroblasts. We suggest that autophagy may be involved in activation of cardiac fibroblasts to myofibroblasts. We show that plating P0 cardiac fibroblasts on stiff plastic substrata induces the myofibrobast phenotype, concomitantly with the induction of autophagy. Inhibition of autophagy with pharmacologic inhibitors represses the myofibroblast phenotype (Figure 6). Further work using autophagy inhibitors is needed to develop new anti-fibrotic therapies to treat the fibrosing heart.

## MATERIALS AND METHODS

### Animal ethics

Experimental protocols involving animals were reviewed and approved by the University of Manitoba's Animal Care Committee following the Canadian Council of Animal Care Standards.

### Cell culture

Cardiac fibroblasts were isolated using retrograde Langendorff perfusion as previously described [49]. Hearts were isolated from male Sprague-Dawley rats (150 to 200 g) and subjected to a 5 minute perfusion with Dulbecco's Modified Eagles Medium: Nutrient Mixture F-12 (DMEM-F12) supplemented with 100 U penicillin, 100 U streptomycin, and 1.0 μM ascorbic acid followed by a 6 minute perfusion with Minimum Essential Medium Eagle Spinner Modification (SMEM). Subsequently, the hearts were perfused with collagenase type II (Worthington, LS004177 ; 0.1% w/v) for 20 minutes, then minced and incubated in dilute collagenase (0.05%w/v) at 37°C for 10 minutes. The collagenase was neutralized by adding 2× volume complete growth media (10% fetal bovine serum (FBS) DMEM-F12). The tissue was then further digested by trituration and filtered through a 70 μm cell strainer (Fisher Scientific) to remove undigested tissue. The filtered tissue suspension was collected in a 50 mL conical tube and centrifuged at 750 × *g* for 7 minutes at room temperature. After centrifugation, the supernatant was discarded and the cell pellet was re-suspended in 10 mL of growth media. Lastly, each cell pellet was diluted to a final volume of 30 mL, and seeded in 100 mm plastic cell culture dishes and allowed to adhere for 2 hours at 5% CO_2_, 37°C. After incubation, cell cultures were washed twice with 1X phosphate-buffered saline (PBS) and fresh 10% FBS DMEM-F12 media was then added. The cultures were returned to the incubator and incubated at 37°C with 5% CO_2_ overnight. The next day, cultures were rinsed once with 1X PBS and fresh media was added. Once the cultures reached 50–60% confluency, they were treated individually with the autophagy inhibitory drugs Baf-A1 (Sigma-Aldrich, B1793) at 5.0 nM, and 7.5 nM or CQ (Sigma-Aldrich, C6628) at 25 μM, 35 μM, and 50 μM for 48 hours.

### Protein isolation

After pharmacological treatment, cells were rinsed once with 1X PBS before being mechanically scraped from the plate in 1.5 mL of ice cold 1X PBS. The cell suspension was collected into a microcentrifuge tube and centrifuged at 16000 × *g* for 5 minutes at 4°C. The supernatant was discarded and the pellet re-suspended in RIPA buffer [1% NP-40, 1.0 mM ethylene glycol-bis (β-aminoethyl ether)-N,N,N',N'-tetraacetic acid (EGTA)] containing phosphatase inhibitors (10 mM sodium fluoride, 1.0 mM sodium orthovanadate) and protease inhibitors (Sigma-Aldrich, P8540). Lysates were incubated on ice for 1 hour, sonicated 3 times for 10 seconds each and centrifuged at 16000 × *g* at 4°C for 15 minutes to pellet cellular debris. The supernatant containing the isolated protein was used for protein quantification using the bicinchoninic acid method [50].

### Western blot analysis

SDS-PAGE of 10–20 μg of protein was performed on 6-12% (non-gradient) gels. Proteins were transferred to polyvinylidene difluoride (PVDF) membranes and blocked in PBS or Tris-buffered saline (TBS) with 0.1% Tween-20 containing 10% (w/v) skim milk for 1.5 hours at room temperature with constant shaking. Primary antibodies were diluted in PBS with 3% (w/v) skim milk or 5% (w/v) bovine serum albumin (BSA) according to the manufacturer's protocol [α-SMA (1:5000;Sigma, A2547), EDA-fibronectin (1:1000; Millipore, MAB1940), LC-3β (1:2000; Sigma, L7543) or β-tubulin (1:5000; Abcam, ab6046); eEF2 (1:3000;Cell Signalling, 2332), p62 (1:1000; Cell Signalling, 5114), total-p38(1:1000; Cell Signalling, 9212), and phospho-p38 (1:1000; Cell Signalling, 9211)]. Membranes were incubated with primary antibodies overnight at 4°C with constant shaking. After incubation, the membranes were washed three times for 10 minutes each in PBS-T/TBS-T, followed by incubation with secondary antibodies (1:10,000) of either HRP-labelled goat anti-rabbit (Jackson Immuno Research, 111-035-003) or goat anti-mouse antibodies (Jackson Immuno Research, 115-035-003) in 3% (w/v) skim milk PBS-T or 1% (w/v) BSA in TBS-T for 1.5 hours at room temperature with shaking. Following the secondary incubation, the blots were washed 3–4 times in PBS-T/TBS-T. Protein bands were visualized using ECL or ECL Plus (Thermo Scientific, 34080) and developed on X-ray film. Equal protein loading was confirmed using Eukaryotic Elongation Factor 2 (eEF2) or β-tubulin by immunoblotting. Films were scanned and digitized using a GS-800 Densitometer (Bio-Rad) and bands were quantified using Quantity One (Bio-Rad) software, with pixel saturation and automated band identification.

### Immunofluorescence

P0 rat cardiac fibroblasts were seeded onto six-well tissue culture plates containing glass cover slips. Cells were allowed to adhere and reach 50–60% confluency before being treated with Baf-A1 (5.0 nM, and 7.5 nM) or CQ (25 μM and 50 μM) for 48 hours. The cells were fixed with 4% paraformaldehyde (PFA) for 15 minutes at room temperature, followed by 15 minutes of permeabilization with 0.1% Triton X-100 in 1X PBS. Cells were washed with 1X PBS and incubated overnight with anti-α-SMA and anti-LC-3β primary antibodies (1:500) in 1% (w/v) BSA in PBS at 4°C. The next day, cells were washed three times for 15 minutes each and then incubated with fluorescently-labelled secondary antibodies (Alexa Fluor 555 goat-anti mouse (Invitrogen, A21422), 1:700 and Alexa Fluor 488 donkey anti-rabbit (Invitrogen, A21206), 1:700) for 90 minutes at room temperature. Cells were washed again three times for 15 minutes each. Cover slips were mounted onto glass slides using Prolong^®^ Gold anti-fade reagent with DAPI (Life Technologies, P36962). Images were acquired using a Zeiss Axiovert 200M epifluorescence microscope and analyzed with Axio vision software (Carl Zeiss Microsopy GmbH).

### Transmission electron microscopy (TEM)

Cells were fixed in Karnovsky fixative and the pellet was resuspended in 5% sucrose in 0.1 M Sorensen's phosphate buffer overnight at 4°C. Cells were pelleted, post-fixed with 1% osmium tetroxide in 0.1 M Sorensen's buffer for 2 hours, and then dehydrated and embedded in Embed 812 resin for sectioning. First, semi-thin sections (1 μM) were cut from the blocks and stained with toluidine blue for inspection. Next, thin sections (200 nM) were cut, and placed on copper grids for staining with uranyl acetate and counter staining with lead nitrate. Imaging was done using a Philips CM10 electron microscope.

### Scratch assay

P0 rat cardiac fibroblasts were used for cell scratch/wound healing assays. Silicone inserts (Ibidi, Martinsried, Germany) were placed in a 6-well culture dish and a cell suspension of 2.85 × 10^9^ cells/mL was prepared. 70 μL of this cell suspension (approximately 2.5 × 10^5^ cells) was added to each chamber of the silicone insert separated by the silicone divider of the insert. The area surrounding the insert was covered with 1.5 mL DMEM-F12 media to prevent drying out of cells. Cells were allowed to adhere and grow overnight in complete growth media (10% FBS DMEM-F12). The next day, the media was changed to a 2% FBS DMEM-F12 solution and cells were incubated in low serum for an additional 24 hours. Cells were then treated with Baf-A1 (7.5 nM) or CQ (35 μM) for 48 hours. After 48 hours, inserts were removed and light microscopy images acquired every 6 hours until the untreated control cells reached 100% confluency (i.e. cell migration was complete). Data were analyzed by comparing the difference between the scratch area percentage at 0, 12 and 24 hours in Baf-A1 and CQ treated cells versus controls using Wimasis Image Analysis software (Wimasis Image Analysis, Munich, Germany).

### Gel contraction assay

Collagen gels were prepared by mixing 7.0 mL of a cold (4°C) Type I Bovine Collagen Solution (Advanced Biomatrix, 5005-B) with 2.0 mL of 5X concentrated DMEM-F12, supplemented with 100 U penicillin, 100 U streptomycin and 1.0 μM ascorbic acid (pH 7.4). The final volume was adjusted to 10 mL with distilled water. Gels were cast in a 24-well cell culture plate by adding 600 μL of the gel solution to each well. The gels were allowed to solidify overnight at 37°C in a 5% CO_2_ incubator. Freshly-isolated P0 rat cardiac fibroblasts were then seeded onto the gels. Approximately 2.0 × 10^4^ cells were plated in each well with 10% FBS DMEM-F12 media and allowed to adhere for 2 hours. Cells were rinsed twice with 1× PBS followed by adding fresh complete growth media and allowed to grow for 24 hours. Cells were then cultured in low serum media (2% FBS DMEM-F12) for the remaining experimental time. Following 24-hour incubation in low serum media, gels were released from the wells using a circular cutting tool. Cells were then treated with TGF-β_1_ (10 ng/mL; Cell Signaling; positive control group), CQ (10, 25 and 35 μM), Baf-A1 (7.5 nM), CQ (10 μM, 25 μM and 35 μM) + TGF-β_1_ (10 ng/mL), Baf-A1 (7.5 nM) + TGF-β_1_ (10 ng/mL). Untreated cells served as control groups. Images were taken immediately following treatment (*t* = 0 hours), and 24, 48 and 72 hours post-treatment. Gels were analyzed using IDL Measure Gel software (University of Calgary, AB, Canada) to determine total gel surface area at each time point.

### Statistical analysis

Data are expressed as mean ± SEM. Each n represents a single animal, and all studies were conducted with at least 3 individual animals. Statistical significance between the groups was assessed by one-way ANOVA followed by a Student-Newman-Keuls post-hoc test using SigmaPlot software (Systat Software, San Jose, CA, USA). A *P*-value < 0.05 was considered statistically significant.
